# Determination of Per- and Polyfluoroalkyl Substances in Craft Villages and Industrial Environments of Vietnam

**DOI:** 10.1155/2021/5564994

**Published:** 2021-04-21

**Authors:** Thi Vi Phung, Thuy Ngoc Nguyen, Lan-Anh Phan Thi, Hung Viet Pham, Hong Anh Duong

**Affiliations:** ^1^Key Laboratory of Analytical Technology for Environmental Quality and Food Safety Control, VNU University of Science, Vietnam National University, Hanoi 334 Nguyen Trai Thanh Xuan, Hanoi 100000, Vietnam; ^2^Research Centre for Environmental Technology and Sustainable Development, VNU University of Science, Vietnam National University, Hanoi 334 Nguyen Trai Thanh Xuan, Hanoi 100000, Vietnam

## Abstract

Per- and polyfluoroalkyl substances (PFASs) have attracted great concern because of their great recalcitrant nature and harmful environmental health effects. Eight PFASs in wastewater from craft villages and industrial environments of Vietnam were analyzed using liquid chromatography triple quadrupole mass spectrometry (LC-MS/MS) with negative electrospray ionization interface. For analysis of PFASs, percent recoveries ranged from 87 to 112, and MQL varied from 0.19 ng/L to 0.49 ng/L. Treated wastewater samples from eight metal-plating and eight textile-dyeing factories were collected for analysis of PFASs. Concentrations of PFOS in wastewater samples obtained from metal-plating factories with decorative plating stage were found at a range of 0.73–18.91 ng/L. For textile-dyeing factories, PFOA and/or PFHxA, which were present in all effluent wastewater samples, varied from 0.37 to 15.96 ng/L and 1.07 to 43.58 ng/L, respectively. Sixty surface water samples in four locations of the textile dyeing craft villages, a recycling plastic village, a paper recycling village, and 10 river water samples in the control area (a rural area without specific waste sources) were collected and analyzed for PFASs. The total concentrations of eight PFASs in surface water samples of craft villages ranged from 0.83 to 58.2 ng/L, which were significantly higher than those in the control area. PFOA, PFHxA, and PFOS are the three most dominant congeners in wastewater taken from craft villages with the highest concentrations of 27.4, 23.8, and 7.36 ng/L, respectively. The environmental risks posed by PFASs in surface water from craft villages were mainly in a range of extremely low to low level, particularly a few points have high ecological risks of PFDoA.

## 1. Introduction

Per- and polyfluoroalkyl substances (PFASs) comprise a large group of compounds that include perfluoroalkyl acids (PFAAs) and those categorized as perfluoroalkyl carboxylic acids (PFCAs) and perfluoroalkyl sulfonic acids (PFSAs) [1]. These compounds include single or multiple aliphatic chains for which some or all hydrogen atoms attached to carbon atoms have been replaced by fluorine (polyfluorinated or perfluorinated, respectively) [2]. Due to their lipophobic and hydrophobic properties, they have been widely used for more than 60 years in numerous industrial and consumer applications including lubricants, fire-fighting foams, electroplating additives, textile coatings, carpets, packing papers, cosmetics, and cleaners [3]. As a consequence of the widespread use and extreme resistance to degradation, PFASs have been globally found in the environment (e.g., water, soil, and sediment), wildlife, and humans [1, 3–6]. Among PFASs, perfluorooctane sulfonate (PFOS) and perfluorooctanoic acid (PFOA) have gained more attention than other compounds because of their potential adverse effects on aquatic ecosystems and human health. For this reason, PFOA and PFOS were added in the Stockholm Convention on Persistent Organic Pollutants (POPs) in 2009 and 2015, respectively [7, 8]. As a class of POPs, exposure to PFASs may lead to significant harmful effects on immunologic and metabolic functions, reproduction, neurodevelopment, and children development [9].

In recent decades, the investigation of PFASs in the aquatic environment has been well documented in the world, especially in developed countries. The concentrations of PFASs have varied from nanograms to micrograms per liter in surface water and wastewater worldwide [10, 11]. One of the major sources of PFASs is waste streams from wastewater treatment plants (WWTP), which have been detected in high levels. Kim et al. (2021) measured PFASs in wastewater effluents from eight different types of industrial factories in Korea and detected 19 target compounds with a mean PFAS concentration of 5.18 *μ*g/L [12]. Another study showed that total concentrations of 11 PFASs in wastewater from WWTPs in Japan ranged from 50.4 to 2342.9 ng/L [13]. Kunacheva et al. reported the high level of 10 PFASs was detected in WWTPs in Thailand ranging between 674–1383 ng/L [14]. Among PFASs, PFOA and PFOS are the two most frequently observed PFASs in the aquatic environment. However, PFOA and PFOS can now only be used in few applications, so short-chain PFASs such as fluorotelomers and perfluoropolyethers have been developed to replace these compounds. A number of countries have replaced long-chain PFASs since 2000 by using perfluorobutane sulfonate (PFBS) and perfluorobutanoic acid (PFBA). Recent studies indicated that the concentration of long-chain PFASs in WWTPs is often much lower than that of the short-chain PFASs [15].

According to the UNEP guidance for the inventory of PFOS and related chemicals, PFASs have various specific uses as a chemical agent in industrial sectors such as electronics, metal plating, paper, and textiles. They may also be present in consumer products under the group: textiles, upholstery, carpets, leather, paper and food paper packing, and synthetic detergents or specialized products such as fire fighting foams, aviation hydraulic fluids, and surfactants used in metal plating [16]. Therefore, during the manufacturing process, PFASs can enter wastewater and solid waste and even enter the aquatic environment if they are not properly treated. On the other hand, consumer products will probably be a source of PFASs into the environment if these products after being used are improperly discarded, recycled (paper, plastic, electronics, etc.), or buried. Similar to other developing countries, the Vietnam government has faced the increasing chemical pollution because of the rapid industrialization level and lack of effective chemical control as well as waste management [17]. In Vietnam, the current status of urban wastewater treatment and wastewater from craft villages and industrial zones is scattered and inadequate. For this reason, wastewater is discharged directly into drainage canals, which eventually entered surface water systems such as lakes, ponds, and rivers. Importantly, PFASs along with effluents can likely enter the aquatic environment and affect the surrounding ecosystems. A few earlier studies indicated the widespread presence of PFASs in municipal wastewater and surface water near waste recycling sites, disposal sites, and wastewater treatment plants in Vietnam [17, 18]. However, there is no research on occurrence of PFASs in wastewater from craft villages and industrial factories, which have the high potential of PFASs contamination.

In recognition of these concerns, the current study is aimed at determining contamination levels of PFASs and evaluating potential wastewater sources like wastewater from weaving and dyeing, paper recycling, plastics recycling villages, metal plating, and textile factories. Occurrence data of PFASs then are compared with the results of previous studies, and the possible risks of PFASs to aquatic organisms are assessed based on the measured environmental concentration and ecological toxicity to aquatic ecosystems.

## 2. Materials and Methods

### 2.1. Chemicals and Reagents

In this study, eight PFASs standards and labeled surrogates were purchased from Wellington Laboratories (Ontario, Canada), with purities of >99%. Standard reagents include two perfluoroalkyl sulfonates (perfluorohexanesulfonate (PFHxS) and perfluorooctylsulfonate (PFOS)) and six perfluoroalkyl carboxylates (perfluorohexanoic acid (PFHxA), perfluorooctanoic acid (PFOA), perfluorononanoic acid (PFNA), perfluorodecanoic acid (PFDA), perfluoroundecanoic acid (PFUdA), and perfluorododecanoic acid (PFDoA)). PFASs stock solution (2000 ng/mL) was prepared by mixing perfluoroalkyl sulfonates solution and perfluoroalkyl carboxylates solution into methanol and stored at 4^o^C. Working standard solutions were prepared using different dilutions of the mixed standard solution with methanol. Eight ^13^C-labeled PFASs (i.e., ^13^C_6_-MPFHxA, ^13^C_8_-MPFOA, ^13^C_9_-MPFNA, ^13^C_10_-MPFDA, ^13^C_11_-MPFUnA, ^13^C_12_-MPFDoA, ^13^C_6_-MPFHxS, and ^13^C_8_-MPFOS) were used as surrogates (SRs). SRs (2.5 ng/mL) were prepared by mixing into methanol and stored at 4°C. All samples were spiked with SRs at 2.5 ng/mL with a volume of 2.5 *μ*L to find their recoveries. Oasis WAX solid phase extraction cartridges were purchased from Waters (MA, USA) and used to concentrate samples prior to analysis. Ammonium acetate, ammonia hydroxide, acetic acid, and methanol (MeOH) were obtained from Sigma-Aldrich (USA). These chemicals were used to prepare the mobile phases and elution solutions.

### 2.2. Sample Collection

In craft villages, wastewater samples were surface water samples that obtained from drainage canals, ponds, and rivers receiving wastewater from production workshops. In this study, surface samples were taken from canals near textile dyeing workshops in Ha Tay craft villages, i.e., Van Phuc and La Khe villages (Hanoi city, HT1-HT15), Hoi Quan village (Bac Ninh Province, HQ1-HQ15), paper recycling workshops in Phong Khe village (Bac Ninh Province, PK1-PK15), and plastics recycling workshops in Nhu Quynh village (Hung Yen province, HY1-HY15); the number of each sampling site was 15. With a total length of more than 200 km, the Day River which flows through rural areas without potential source of PFASs pollution was selected as a control area. The number of water river samples was 10.

Moreover, effluent wastewater samples were taken from the selected industrial factories including metal plating factories (*n* = 8) and textile dyeing factories (*n* = 8), which have the high potential of PFASs emissions.

Precleaned glass bottles with a capacity of 500 mL were used for collection of surface water samples. Samples were obtained at each site from 50 cm depth below the surface and were placed in coolers on ice during transportation. Wastewater samples were collected in selected industrial factories after the treatment process. All samples were stored at 4°C until analyzed.

### 2.3. Sample Pretreatment

The water sample was thawed at room temperature (20°C) on the day of extraction. Analysis of water was conducted according to the standard entitled “Water quality—Determination of perfluorooctanesulfonate (PFOS) and perfluorooctanoate (PFOA)—method for unfiltered samples using solid phase extraction and liquid chromatography/mass spectrometry” - ISO 25101 : 2009 with minor modifications [19]. Briefly, the water sample (1 L) was filtered through a 47 mm glass fiber filters to separate suspended solids. Then, the sample was spiked with known amounts of isotope-labeled SRs to quantify procedural recovery. An Oasis WAX cartridge was preconditioned with 4 mL of MeOH containing 0.1% ammonia followed by 4 mL of MeOH and 4 mL of deionized water. The water sample was then loaded through a WAX cartridge with a flow rate of 10 mL/min. The SPE cartridge was washed with 4 mL of 25 mM ammonium acetate and dried for 15 min. Next, the target compounds in the cartridge were eluted with 4 mL of MeOH and 4 mL of 0.1% ammonia solution at a rate of 1 drop per second. The extract was concentrated to 1 under a gentle nitrogen stream. A 0.2 *μ*m nylon membrane filter was used to filter the final eluate before LC-MS/MS analysis.

### 2.4. Instrumental Analysis

For PFASs analysis, the 2 *μ*L samples were injected into a liquid chromatography tandem triple-quadrupole mass spectrometer (LC-MS/MS; Shimadzu, Japan). Chromatographic separation of PFASs was performed on a Poroshell 120 EC C18 column (2.1 mm × 150 mm, 2.7 *μ*m) and a guard column EC C18 (Agilent, USA). Separation of PFASs was achieved with the mobile phase of 2 mmol/L ammonium acetate in methanol (9 : 1, v/v) (A) and methanol (B) under a gradient elution program. The program was performed as follows: 0–2 min, 10%–30% B; 3–22 min, 30%–95% B; 23–25 min, 30% B. The column temperature was 40°C, and the flow rate was 0.3 mL/min. The DL and heat block temperatures were hold at 250°C and 400°C, respectively. The flow rates of the drying gas and nebulizing gas were 15 L/min and 3 L/min, respectively. Mass spectrometry analysis of eight PFASs was performed in negative electron ionization mode and the multiple reaction monitoring (MRM).  Table 1 shows the analytical parameters of each PFAS.

### 2.5. Quality Control and Method Performance

Six-point calibration curves were developed for each target analyte by diluting calibration stock in methanol at concentrations ranging from 0.5 to 20 ng/mL. To assess procedural and matrix loss of water samples during the experimental procedure, the procedural blanks and matrix spikes were analyzed after every ten samples. The signal-to-noise ratios of 3 and 10 were defined as limits of detection (LOD) and limits of quantification (LOQ), respectively. Based on the visual inspection of the chromatograms, all samples below the LOD were clearly nondetectable. The relative standard deviations (RSDs) were in a range of 4–17%. The recoveries of eight PFASs in water samples ranged from 87% to 112%. The method detection limits (MDLs) of target PFASs were in a range of 0.19–0.49 ng/L.

### 2.6. Environmental Risk Assessment of PFASs

The environmental risk of the PFASs was assessed based on their risk quotient (RQ) calculated by dividing the measured concentration of the target substance (MEC) with the predicted no-effect concentration (PNEC) [20]:

(1) $$\begin{array}{c} \text{RQ}=\frac{\text{MEC}}{\text{PNEC}}. \end{array}$$

The RQ value exceeds 1.0 means that the corresponding pollutant is high risk. The RQ values in the range of 0.1–1.0 and 0.01–0.1 represent medium and low risk, respectively.

## 3. Results and Discussion

### 3.1. Occurrence of PFASs in Wastewater from Factories with Metal-Plating Stage and Textile-Dyeing Factories

The concentrations and profiles of eight target PFASs in wastewater from factories are presented in Table 2. For factories with metal plating stage, PFOS was frequently used as a component with 5–10% content in intermediate substances such as surfactants, wetting agents, and mist inhibitors [21]. Therefore, PFOS was probably released into wastewater from factories during the production process. Five wastewater samples collected from the metal plating plants for furniture had the highest PFOS levels in the range of 5.41–18.91 ng/L followed by wastewater of electronics plating factories with a concentration of 0.73 ng/L. In contrast, PFOS was completely absent in samples from three steel plants with the galvanizing stage.

In the case of wastewater from textile factories, only PFOA and PFHxA were detected. Specifically, PFOA was present in all samples, while PFHxA was observed with a detection frequency of 75%. The concentrations of PFOA and PFHxA ranged from 0.37 to 15.96 ng/L and from 1.07 to 43.58 ng/L, respectively. In the past, C8 PFASs were previously used for the surface treatment stage of fabric products to give textiles water-, dirt-, and oil-repellent properties. In 2013, PFOA compounds were also identified as a chemical of very high concern and were added to the REACH (EU Chemicals Regulation) candidate list. According to EU Regulation 2017/1000, PFOA and precursor compounds (i.e., substances that can release PFOA) may not be produced or placed on the EU market after 4 July 2020. For certain areas of application such as occupational safety textiles or membranes for medical textiles, an extended deadline applies until 2023 [22]. C6 fluorocarbon resins are now increasingly being used as an alternative to C8 chemistry. However, a restriction or even prohibition procedure similar to those for PFOA is already emerging. This most recent proposition for restricting C6 use within Europe is expected to be filed in Q3 of the year 2020 and comes after similar German-led efforts were knocked back in November 2018. The analysis results in this study (with a sampling time of 2019) revealed that besides the C6 PFASs, there is still use of C8 compounds in the surveyed textile factories.

### 3.2. Concentrations and Profiles of PFCs in Surface Water from Craft Villages

**Figure 1:**
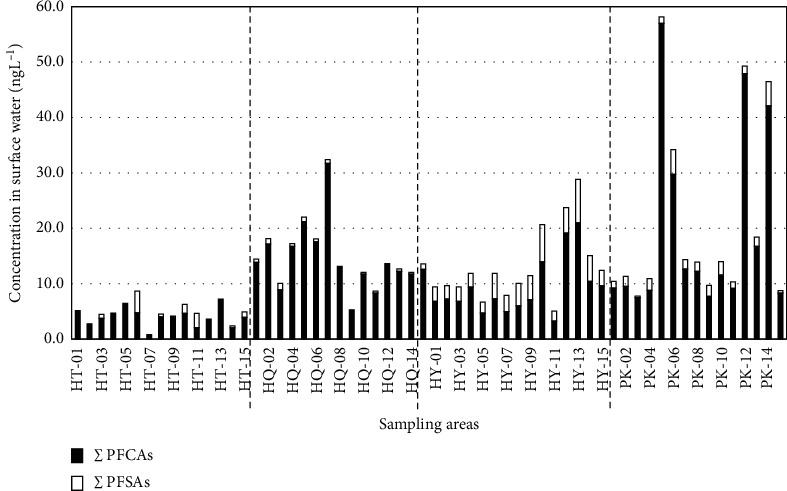
Total concentrations of PFCAs and PFSAs in water samples taken from craft villages.

Sixty surface samples were collected from canals that received directly untreated wastewater from four craft villages including the textile dyeing, paper recycling, and plastics recycling villages. The concentration range and median concentration of PFASs in each location are summarized in Table 3 and Figure 1. Concentrations of PFASs in surface water samples from craft villages were presented in Table S1 in Supplementary Information. Analysis results indicated that all PFASs were detected in surface water at the following frequencies: PFOA (100%), PFHxA (98%), PFOS (80%), PFDA (58%), PFNA (52%), PFHxS (20%), and PFDoA and PFUnDA (8%). The concentration of individual (median and range, in ng/L) decreased in the order: PFOA (3.43; 0.83– 27.4), PFHxA (2.64; <0.26–23.8), PFOS (0.90; <0.19–7.36), PFDA (0.52; <0.36–2.63), PFNA (0.32; <0.41–7.24), PFHxS (<0.31; <0.31–0.98), PFDoA (<0.29; <0.29–11.92), and PFUnDA (<0.49; <0.49–1.96). Like municipal surface water in Vietnam, the occurrence of PFOA, PFHxA, and PFOS was common. There was a similar phenomenon in abundance of PFNA in surface water receiving wastewater in Vietnam (this study, [18]), Japan [20, 21], and China [22–24], possibly due to the massive use of products originating from the two first countries. This abundance was different from the results of other studies in Thailand [23] or European countries.

The total concentration of eight target PFASs in surface water of craft villages ranged from 0.83 to 58.2 ng/L with a median value of 12.4 ng/L. The presence of PFCAs in surface water was dominant to PFSAs in concentration and frequency, which can be explained by the better solubility of PFCAs and the different characteristics of waste sources in craft villages. Concentrations of PFASs in surface water samples from the Day River (ng/L) to the control area are indicated in Table S2 in Supplementary information. The *t*-test illustrated that the total concentration of eight PFASs in each group of trade villages was statistically significantly different from those in the Day River to the control area (the rural region with none of the specific sources of PFASs pollution is shown in Table 2 in Supplementary Information) (*p* < 0.05). This result proved that the above groups of craft villages are potential contamination sources of PFASs.

### 3.3. Concentrations of PFASs in Craft Villages in comparison with Those in Other Studies

Total concentrations of PFASs, PFCAs, and PFSAs in water samples collected from different groups of craft villages are compared in Table 4. It is obvious that the PFASs contamination level in the surface water of the paper recycling village (Phong Khe) was the highest (13.9; 7.74–58.2) followed by textile village (Hoi Quan, 13.4; 5.30–32.4), plastics recycling village (Nhu Quynh, 11.5; 5.06–28.8), and the lowest concentration was in Ha Tay (textile, 4.67; 0.83–8.65). On the other hand, there were differences in the profiles of PFASs in surface water between studies sites. While the PFSA concentrations in the textile areas were very low, this group of compounds was determined at a much higher concentration in the plastics and paper recycling area. The second one also had the highest detected frequencies for all PFASs.

In the comparison between surface water receiving wastewater from production activities of textile villages and textile factories, the occurrence of more congeners was found in samples of textile villages. That might be explained due to mixed wastewater from potential sources for PFASs such as domestic activities, dumping sites, or other small production activities. Despite the same textile group, the higher scale and capacity of the production in Hoi Quan village compared with Ha Tay villages might lead to a higher level of PFASs contamination in surface water there. In fact, Phong Khe (Bac Ninh) and Nhu Quynh (Hung Yen) are two regions with the largest paper and plastic recycling activities in Northern Vietnam. The above-mentioned results reflected that the potential for PFASs contamination in surface water from trade villages depended on the production characteristics, the use of chemicals or materials containing PFASs, as well as their scale production.

Concentrations of PFOS and PFOA in surface water from textile, paper recycling, and plastics recycling areas with large-scale production were two to five times higher than those in surface water receiving urban wastewater in big cities and five to ten times greater than those in big rivers flowing through many cities or rural areas. In comparison to the published results in Asia, these concentrations are similar to those found in river water in Thailand, Malaysia, and South Korea but lower than surface water in Japan, Singapore, and China.

### 3.4. Environmental Risk of PFASs

The PNEC_water_ of PFOS, PFOA, and others PFASs are recommended as listed in Table 5 according to several publications [30–33]. The RQ_water_ of PFHxA, PFNA, and PFHxS were < 0.01 for all surface water samples, which indicated extremely low environmental risk. The RQ_water_ of PFOA, PFDA, and PFOS ranging from < 0.01 to <0.1 means the environmental risk levels of PFOA, PFDA, and PFOS varied from extremely low to low. It is noted that two PFCAs with long aliphatic chains including PFUdA and PFDoA showed the higher toxicity than short-chain compounds at a few locations in textile dyeing villages; RQ_water_ represented the immediate or even high risk. The concentrations of PFASs in surface water from these craft villages were further compared with the environmental quality standards for surface water regulated by the European Commission through Directive 2013/39/EU (Directive 2013/ 39/EU) [34]. In this study, the average concentration of PFOS (1.60 ng/L) was higher than the annual average environmental quality standard (AA-EQS; 0.65 ng/L) of PFOS for inland surface water and lower than the maximum allowable concentration (MAC-EQS; 36 mg/L) (Directive 2013/39/EU) [34]. It can be seen that wastewater from a number of craft villages were the PFOS release sources into the environment; surrounding surface water need to be under the sufficient control and management.

## 4. Conclusions

To the best of our knowledge, this is the first study in Vietnam to investigate the presence of PFASs in the aquatic environment of selected potential production activities for PFASs contamination, i.e., textile dyeing craft villages, recycling plastic and paper villages, and textile and metal plating factories. The occurrence of PFOA, PFHxA, and PFOS ranging from sub- to tens of ng/L in treated wastewater from textile and metal plating factories suggested that these compounds have still been used although C8 was banned in textile production. This finding revealed lack of effective wastewater treatment for this chemical class. In craft villages, analysis of PFASs in surface water receiving untreated wastewater sources from both production and domestic activities showed that PFOA, PFHxA, and PFOS were observed in all samples with concentrations ranging from sub- to tens of ng/L. Average concentrations of these compounds were two to ten times greater in surface water obtained from the control area as well as municipal surface water. Concentrations and detection frequencies (more than 50%) of five PFASs in surface water samples from craft villages decreased in the following order: PFOA > PFHxA > PFOS > PFDA > PFNA. The results of the study indicated that the average concentration of PFOS in surface water from craft villages was higher than the annual average environmental quality standard under the European Commission. The environmental risks posed by PFASs in surface water from craft villages were mainly in a range of extremely low to low level for PFASs with carbon chain length shorter than 10, particularly a few points have high ecological risks of PFUdA and PFDoA. It is confirmed that production activities in craft villages such as textile dyeing craft villages, recycling plastic, and paper villages are significant sources of PFASs into the aquatic environment, which should be controlled in Vietnam.

## Figures and Tables

**Table 1:** MS/MS parameter for PFAS analysis.

No.	PFAS	Precursor ion (m/z)	Product ion (m/z)	Q1 Pre Bias (V)	CE (V)	Q3 Pre Bias (V)
1.	MPFHxS	403	73.9/102.9	19/19	49/39	28/15
2.	MPFOS	503	79.9/99.1	24/24	55/48	29/15
4.	MPFHxA	314.95	270.15/119.15	15/15	8/20	25/20
5.	MPFOA	416.95	372.05/172.2	20/20	10/19	23/30
6.	MPFNA	467.95	423.1/219.15	22/22	10/16	26/20
7.	MPFDA	514.9	469.95/219.10	24/24	11/19	30/19
8.	MPFUdA	564.9	519.95/169.1	28/28	11/26	34/28
9.	MPFDoA	614.9	569.9/169.1	30/30	12/30	36/28
10.	PFHxA	312.8	269/118.95	22/22	9/21	25/18
11.	PFOA	412.8	368.95/169.05	20/20	10/19	22/28
12.	PFNA	462.8	418.95/219.05	22/22	10/17	26/20
13.	PFDA	512.85	469.2/219.1	24/24	11/19	30/12
14.	PFUdA	562.8	518.95/269.1	40/40	12/17	34/26
15.	PFDoA	612.8	568.95/318.75	22/22	12/20	38,29
16.	PFHxS	398.8	79.95/98.95	27/27	46/35	28/16
17.	PFOS	498.85	80.15/99.05	24/24	50/43	28/15

**Table 2:** Concentrations of PFASs (ng/L) in wastewater of selected metal-plating factories and textile-dyeing factories in Vietnam.

Name	PFHxA	PFOA	PFNA	PFDA	PFUdA	PFDoA	PFHxS	PFOS	Σ8 PFCs	ΣPFCAs	ΣPFSAs
Metal-plating factories											
W98	NQ^a^	2.19	NQ	1.24	NQ	NQ	NQ	18.9	22.3	3.43	18.9
BG01	0.68	0.58	NQ	0.53	NQ	NQ	1.26	5.41	8.46	1.79	6.67
BG02	0.92	0.5	NQ	0.86	NQ	NQ	0.88	8.64	11.8	2.28	9.52
BG03	0.70	0.96	NQ	0.59	NQ	NQ	NQ	17.7	19.9	2.25	17.7
BG04	0.45	1.03	NQ	NQ	NQ	NQ	NQ	0.73	2.21	1.48	0.73
W3	NQ	0.37	NQ	NQ	NQ	NQ	NQ	NQ	0.37	0.37	0
W97	NQ	0.72	NQ	1.78	NQ	NQ	NQ	NQ	2.5	2.5	0
W96	NQ	NQ	NQ	NQ	NQ	NQ	NQ	NQ	0	0	0
Textile-dyeing factories											
W120	1.14	3.53	NQ	NQ	NQ	NQ	NQ	NQ	4.67	4.67	0
W127	2.09	6.69	NQ	NQ	NQ	NQ	NQ	NQ	8.78	8.78	0
W129	1.07	0.37	NQ	NQ	NQ	NQ	NQ	NQ	1.44	1.44	0
W131	43.6	16.0	NQ	NQ	NQ	NQ	NQ	NQ	59.5	59.5	0
W132	30.1	0.52	NQ	NQ	NQ	NQ	NQ	NQ	30.6	30.6	0
W134	NQ	1.47	NQ	NQ	NQ	NQ	NQ	NQ	1.47	1.47	0
W137	3.18	0.55	NQ	NQ	NQ	NQ	NQ	NQ	3.73	3.73	0
W138	NQ	1.75	NQ	NQ	NQ	NQ	NQ	NQ	1.75	1.75	0
MQL	0.26	0.35	0.41	0.36	0.49	0.29	0.31	0.19			

^a^Not quantified (concentration lower than MQL and treated as 0 in Σ8 PFCs, ΣPFCAs, and ΣPFSAs).

**Table 3:** Concentrations (median and range, ng/L) of PFASs in surface water from selected craft villages in Vietnam.

Sampling areas (number of samples)	PFHxA	PFOA	PFNA	PFDA	PFUDA	PFDoA	PFHxS	PFOS	Σ8 PFASs
*Textile-dyeing villages*									
Van Phuc (Ha Noi) (*n* = 15)	1.14 (<0.26–1.42)	2.04 (0.83–3.48)	<0.41	<0.36 (<0.36–1.90)	<0.49	<0.29 (<0.29–1.44)	<0.31	<0.19 (<0.19–3.91)	4.67 (0.83–8.65)
Hoi Quan (Bac Ninh) (*n* = 15)	9.75 (3.24–17.8)	3.20 (3.27–4.66)	<0.41 (<0.41–3.56)	<0.36 (<0.36–1.52)	<0.49	<0.29 (<0.29–11.92)	<0.31 (<0.31–0.77)	0.38 (<0.19–1.00)	13.4 (5.30–32.4)
*Paper recycling villages*									
Phong Khe (Bac Ninh) (*n* = 15)	3.16 (5.21–23.8)	4.97 (3.15–27.4)	2.26 (1.05–7.24)	1.19 (<0.36–2.43)	<0.49	<0.29	<0.31 (<0.31–0.98)	1.66 (0.23–3.90)	13.9 (7.74–58.2)
*Plastics-recycling villages*									
Nhu Quynh (Hung Yen) (*n* = 15)	2.32 (0.63–9.72)	4.03 (2.00–8.61)	0.50 (<0.41–3.50)	<0.36 (<0.36–2.63)	<0.49 (<0.49–1.96)	<0.29 (<0.29–0.73)	<0.31 (<0.31–0.50)	2.97 (1.83–7.36)	11.5 (5.06–28.8)
Total concentrations of all samples (*n* = 60)	2.64 (<0.26–23.8)	3.43 (0.83–27.4)	0.32 (<0.41–7.24)	0.52 (<0.36–2.63)	<0.49 (<0.49–1.96)	<0.29 (<0.29–11.92)	<0.31 (<0.31–0.98)	0.90 (<0.19–7.36)	10.68 (0.83–8.2)
Detection frequency (%)	98	100	52	58	8	8	20	80	

**Table 4:** Comparison between concentrations of PFOS and PFOA in surface water samples in Vietnam and other countries.

Location	Sample type	Concentration (ng/L)		Reference
		PFOS	PFOA	
Vietnam-craft villages				
Phong Khe (Bac Ninh) (15)	Surface water in drainage canals, rivers	0.23–3.90 (1.66)	3.15–27.4 (4.97)	Present study
Nhu Quynh (Hung Yen) (15)	Surface water in drainage canals, ponds, river	1.83–7.36 (2.97)	2.00–8.61 (4.03)	Present study
Hoi Quan (Bac Ninh) (15)	Surface water in drainage canals, ponds, river	<0.19–1.00 (0.38)	3.27–4.66 (3.20)	Present study
Ha Tay (Ha Noi) (15)	Surface water in drainage canals, ponds, river	<0.19–3.91 (<0.19)	0.83–3.48 (2.04)	Present study
Vietnam-big cities				
Hanoi, Da Nang, Hue, and Ho Chi Minh cities (28)	River water	<0.05–5.30 (0.27)	0.09–18 (0.78)	[18]
Vietnam – great rivers flow through cities, rural areas				
Red river (4)	River water	<0.05–0.04 (0.01)	0.13–0.4 (0.32)	[25]
Dong Nai River and Saigon River (10)	River water	<0.03–1.62 (0.27)	<0.37–6.39 (1.39)	[25]
Mekong River (12)	River water	<0.03–1.06 (0.13)	<0.37–0.85 (0.50)	[25]
Day River (10)	River water	<0.1	0.55–2.01 (1.38)	Present study
Thailand	River water	ND–33.6	ND–36.8	[23]
Malaysia	River water	0.03–27.80	0.05–18.90	[23]
Korea	River water	0.83–15.07	0.56–8.34	[26]
Japan				
Tokyo Bay	Coastal sea water	0.78–17.0	2.70–63.0	[24]
Yodo River	River water	0.56–67.4	6.63–21.6	[23]
Kin Ki River	River water	0.27–13.2	0.43–29.9	[23]
Singapore	Coastal sea water	0.37–26.5	0.72–184	[23]
China				
Taihu lake	Lake river	10.6–36.7	3.6–394	[27]
Liao River	River water	ND–27.9	ND–6.6	[27]
Guangzhou River	River water	0.85–13	0.90–99	[28]
Hanjiang River	River water	ND–88.9	ND–256	[29]

ND: no detection.

**Table 5:** Risk quotients of eight target compounds.

Compounds	PNEC_water_ (ng/L)^a^	RQ			
		Ha Tay villages *Textile dyeing*	Hoi Quan village *Textile dyeing*	Nhu Quynh village *Plastic recycling*	Phong Khe village *Paper recycling*
PFHxA	97,000	<0.01	<0.01	<0.01	<0.01
PFOA	1,428	<0.01	<0.01	<0.01	<0.01 ÷ 0.019
PFNA	1,000,000	<0.01	<0.01	<0.01	<0.01
PFDA	45	<0.01 ÷ 0.042	<0.01 ÷ 0.034	<0.01 ÷ 0.058	<0.01 ÷ 0.054
PFUdA	8	<0.01 ÷ 0.244	<0.01	<0.01	<0.01
PFDoA	1	<0.01 ÷ 1.44	<0.01 ÷ 11.9	<0.01 ÷ 0.725	<0.01
PFHxS	250,000	<0.01	<0.01	<0.01	<0.01
PFOS	610	<0.1	<0.01 ÷ 0.012	<0.01	<0.01

PNEC: predicted no effect concentration; RQ: risk quotient; (a)[30–33].

## Data Availability

The data used to support the findings of this study are included within the article.

## Supplementary Materials

Supplementary Materials Table S1: concentrations of PFASs in surface water samples from craft villages (ng/L). Table S2: concentrations of PFASs in surface water samples from Day River (ng/L) to the control area.
