# Supplementary material for: Determination of Per- and Polyfluoroalkyl Substances in Craft Villages and Industrial Environments of Vietnam
Source: J Anal Methods Chem. 2021 Apr 21;2021:5564994. doi: 10.1155/2021/5564994 (PMC8081634; doi:10.1155/2021/5564994)
Supplement: Supplementary Materials — Table S1: concentrations of PFASs in surface water samples from craft villages (ng/L). Table S2: concentrations of PFASs in surface water samples from Day River (ng/L) to the control area. [file 5564994.f1.docx]

**Supplementary Information**

**Determination of per- and polyfluoroalkyl substances in crafts villages and industrial environments of Vietnam**

Thi Vi Phung,^1^ Thuy Ngoc Nguyen,^2^ Lan-Anh Phan Thi,^1^ Hung Viet Pham^1^ and Hong Anh Duong,^1,2*^

*^1^ Key Laboratory of Analytical Technology for Environmental Quality and Food Safety Control, VNU University of Science, Vietnam National University, Hanoi, 334 Nguyen Trai, Thanh Xuan, Hanoi 100000, Vietnam*

*^2^ Research Centre for Environmental Technology and Sustainable Development, VNU University of Science, Vietnam National University, Hanoi, 334 Nguyen Trai, Thanh Xuan, Hanoi 100000, Vietnam*

Correspondence should be addressed to Hong Anh Duong; duonghonganh@hus.edu.vn

**Table S1.** Concentrations of PFASs in surface water samples from craft villages (ng/L)

**Table S2.** Concentrations of PFASs in surface water samples from Day river (ng/L) – the control area

**Table S1. Concentrations of PFASs in surface water samples from craft villages (ng/L)**

Location: Ha Tay village - Textile and dying

| **No** | **Name** | **HT-01** | **HT-02** | **HT-03** | **HT-04** | **HT-05** | **HT-06** | **HT-07** | **HT-08** | **HT-09** | **HT-10** | **HT-11** | **HT-12** | **HT-13** | **HT-14** | **HT-15** | ***MQL*** |
| --- | --- | --- | --- | --- | --- | --- | --- | --- | --- | --- | --- | --- | --- | --- | --- | --- | --- |
| 1 | **PFHxA** | 1.34 | 1.11 | 1.24 | 1.18 | 1.14 | 1.18 | NQ | 1.36 | 1.14 | 1.18 | NQ | 0.82 | 1.42 | 0.58 | 0.90 | *0.26* |
| 2 | **PFOA** | 1.98 | 1.65 | 2.50 | 2.04 | 3.46 | 1.66 | 0.83 | 2.67 | 3.01 | 3.48 | 1.32 | 1.32 | 2.55 | 1.45 | 3.03 | *0.35* |
| 3 | **PFNA** | NQ | NQ | NQ | NQ | NQ | NQ | NQ | NQ | NQ | NQ | NQ | NQ | NQ | NQ | NQ | *0.41* |
| 4 | **PFDA** | 1.83 | 0.00 | 0.00 | 1.50 | 1.86 | 1.90 | NQ | NQ | NQ | NQ | 0.74 | 0.86 | 1.80 | NQ | NQ | *0.36* |
| 5 | **PFUdA** | NQ | NQ | NQ | NQ | NQ | NQ | NQ | NQ | NQ | NQ | NQ | NQ | NQ | NQ | NQ | *0.49* |
| 6 | **PFDoA** | NQ | NQ | NQ | NQ | NQ | NQ | NQ | NQ | NQ | NQ | NQ | 0.61 | 1.44 | NQ | NQ | *0.29* |
| 7 | **L-PFHxS** | NQ | NQ | NQ | NQ | NQ | NQ | NQ | NQ | NQ | NQ | NQ | NQ | NQ | NQ | NQ | *0.31* |
| 8 | **L-PFOS** | NQ | NQ | 0.72 | NQ | NQ | 3.91 | NQ | 0.51 | NQ | 1.65 | 2.61 | NQ | NQ | 0.36 | 1.00 | *0.19* |
|  | **Σ 8 PFASs** | **5.14** | **2.75** | **4.45** | **4.72** | **6.45** | **8.65** | **0.83** | **4.53** | **4.15** | **6.30** | **4.67** | **3.59** | **7.21** | **2.38** | **4.93** |  |
|  | **Σ PFCAs** | **5.14** | **2.75** | **3.74** | **4.72** | **6.45** | **4.74** | **0.83** | **4.03** | **4.15** | **4.65** | **2.06** | **3.59** | **7.21** | **2.02** | **3.93** |  |
|  | **Σ PFSAs** | **0.00** | **0.00** | **0.72** | **0.00** | **0.00** | **3.91** | **0.00** | **0.51** | **0.00** | **1.65** | **2.61** | **0.00** | **0.00** | **0.36** | **1.00** |  |

Location: Hoi Quan village - Textile and dying

| **No** | **Name** | **HQ-01** | **HQ-02** | **HQ-03** | **HQ-04** | **HQ-05** | **HQ-06** | **HQ-07** | **HQ-08** | **HQ-09** | **HQ-10** | **HQ-11** | **HQ-12** | **HQ-13** | **HQ-14** | **HQ-15** | ***MQL*** |
| --- | --- | --- | --- | --- | --- | --- | --- | --- | --- | --- | --- | --- | --- | --- | --- | --- | --- |
| 1 | **PFHxA** | 8.79 | 13.94 | 5.83 | 13.36 | 17.80 | 10.04 | 11.57 | 7.78 | 3.24 | 9.75 | 5.05 | 6.43 | 10.09 | 9.75 | 4.27 | *0.26* |
| 2 | **PFOA** | 2.75 | 3.20 | 3.07 | 2.95 | 3.41 | 4.65 | 4.66 | 3.94 | 2.06 | 1.98 | 3.27 | 4.46 | 2.14 | 1.98 | 4.59 | *0.35* |
| 3 | **PFNA** | 0.78 | NQ | NQ | NQ | NQ | 0.46 | 3.56 | 0.63 | NQ | NQ | NQ | 2.23 | NQ | NQ | 2.53 | *0.41* |
| 4 | **PFDA** | 1.52 | NQ | NQ | 0.40 | NQ | 0.54 | NQ | 0.76 | NQ | NQ | NQ | NQ | NQ | NQ | 1.18 | *0.36* |
| 5 | **PFUdA** | NQ | NQ | NQ | NQ | NQ | 0.00 | NQ | NQ | NQ | NQ | NQ | NQ | NQ | NQ | NQ | *0.49* |
| 6 | **PFDoA** | NQ | NQ | NQ | NQ | NQ | 1.85 | 11.92 | NQ | NQ | NQ | NQ | NQ | NQ | NQ | NQ | *0.29* |
| 7 | **L-PFHxS** | NQ | NQ | 0.77 | NQ | NQ | 0.56 | 0.47 | NQ | NQ | NQ | NQ | NQ | NQ | NQ | 0.56 | *0.31* |
| 8 | **L-PFOS** | 0.59 | 1.00 | 0.40 | 0.50 | 0.80 | NQ | 0.23 | NQ | NQ | 0.30 | 0.38 | NQ | 0.44 | 0.30 | 0.46 | *0.19* |
|  | **Σ 8 PFASs** | **14.43** | **18.14** | **10.07** | **17.22** | **22.01** | **18.10** | **32.41** | **13.10** | **5.30** | **12.03** | **8.69** | **13.12** | **12.67** | **12.03** | **13.59** |  |
|  | **Σ PFCAs** | **13.84** | **17.14** | **8.90** | **16.71** | **21.21** | **17.54** | **31.71** | **13.10** | **5.30** | **11.73** | **8.32** | **13.12** | **12.23** | **11.73** | **12.57** |  |
|  | **Σ PFSAs** | **0.59** | **1.00** | **1.17** | **0.50** | **0.80** | **0.56** | **0.70** | **0.00** | **0.00** | **0.30** | **0.38** | **0.00** | **0.44** | **0.30** | **1.02** |  |

Location: Nhu Quynh village - Plastic recycling

| **No** | **Name** | **HY-01** | **HY-02** | **HY-03** | **HY-04** | **HY-05** | **HY-06** | **HY-07** | **HY-08** | **HY-09** | **HY-10** | **HY-11** | **HY-12** | **HY-13** | **HY-14** | **HY-15** | ***MQL*** |
| --- | --- | --- | --- | --- | --- | --- | --- | --- | --- | --- | --- | --- | --- | --- | --- | --- | --- |
| 1 | **PFHxA** | 2.04 | 1.81 | 2.32 | 3.55 | 1.89 | 2.12 | 2.01 | 1.74 | 2.44 | 3.20 | 0.63 | 6.05 | 9.72 | 3.76 | 3.51 | *0.26* |
| 2 | **PFOA** | 3.54 | 3.81 | 3.53 | 4.95 | 2.79 | 4.03 | 2.94 | 4.28 | 4.00 | 5.28 | 2.00 | 7.79 | 8.61 | 5.32 | 5.14 | *0.35* |
| 3 | **PFNA** | 0.61 | 0.50 | 0.18 | 0.91 | NQ | NQ | NQ | NQ | 0.70 | 3.50 | NQ | 1.95 | 1.90 | 0.55 | 0.49 | *0.41* |
| 4 | **PFDA** | NQ | 0.48 | NQ | NQ | NQ | 0.47 | NQ | NQ | NQ | NQ | 0.61 | 2.63 | 0.75 | 0.80 | 0.50 | *0.36* |
| 5 | **PFUdA** | 0.64 | 0.62 | 0.81 | NQ | NQ | 0.66 | NQ | NQ | NQ | 1.96 | NQ | NQ | NQ | NQ | NQ | *0.49* |
| 6 | **PFDoA** | NQ | NQ | NQ | NQ | NQ | NQ | NQ | NQ | NQ | NQ | NQ | 0.73 | NQ | NQ | NQ | *0.29* |
| 7 | **L-PFHxS** | NQ | NQ | 0.48 | NQ | NQ | NQ | NQ | NQ | NQ | NQ | NQ | NQ | 0.50 | NQ | NQ | *0.31* |
| 8 | **L-PFOS** | 2.61 | 2.46 | 2.12 | 2.45 | 2.02 | 4.61 | 2.97 | 4.07 | 4.35 | 6.77 | 1.83 | 4.59 | 7.36 | 4.65 | 2.77 | *0.19* |
|  | **Σ 8 PFASs** | **9.42** | **9.67** | **9.42** | **11.86** | **6.70** | **11.88** | **7.92** | **10.09** | **11.48** | **20.70** | **5.06** | **23.72** | **28.84** | **15.08** | **12.40** |  |
|  | **Σ PFCAs** | **6.82** | **7.21** | **6.83** | **9.41** | **4.68** | **7.27** | **4.95** | **6.02** | **7.14** | **13.93** | **3.23** | **19.13** | **20.98** | **10.43** | **9.63** |  |
|  | **Σ PFSAs** | **2.61** | **2.46** | **2.59** | **2.45** | **2.02** | **4.61** | **2.97** | **4.07** | **4.35** | **6.77** | **1.83** | **4.59** | **7.86** | **4.65** | **2.77** |  |

Location: Phong Khe village - Paper recycling

| **No** | **Name** | **PK-01** | **PK-02** | **PK-03** | **PK-04** | **PK-05** | **PK-06** | **PK-07** | **PK-08** | **PK-09** | **PK-10** | **PK-11** | **PK-12** | **PK-13** | **PK-14** | **PK-15** | ***MQL*** |
| --- | --- | --- | --- | --- | --- | --- | --- | --- | --- | --- | --- | --- | --- | --- | --- | --- | --- |
| 1 | **PFHxA** | 2.75 | 2.27 | 2.33 | 2.52 | 23.78 | 5.89 | 3.49 | 3.30 | 0.57 | 3.42 | 1.49 | 13.21 | 3.16 | 7.69 | 2.29 | *0.26* |
| 2 | **PFOA** | 3.28 | 3.83 | 3.15 | 3.40 | 24.73 | 15.41 | 4.97 | 4.86 | 4.35 | 5.17 | 6.08 | 27.44 | 7.94 | 26.25 | 3.26 | *0.35* |
| 3 | **PFNA** | 2.07 | 2.26 | 1.05 | 1.80 | 7.24 | 5.98 | 2.87 | 2.51 | 1.78 | 1.58 | 1.60 | 6.24 | 4.35 | 6.53 | 2.03 | *0.41* |
| 4 | **PFDA** | 1.10 | 1.19 | 0.98 | 1.10 | 1.24 | 2.43 | 1.30 | 1.55 | 1.02 | 1.37 | NQ | 1.03 | 1.26 | 1.61 | 0.71 | *0.36* |
| 5 | **PFUdA** | NQ | NQ | NQ | NQ | NQ | NQ | NQ | NQ | NQ | NQ | NQ | NQ | NQ | NQ | NQ | *0.49* |
| 6 | **PFDoA** | NQ | NQ | NQ | NQ | NQ | NQ | NQ | NQ | NQ | NQ | NQ | NQ | NQ | NQ | NQ | *0.29* |
| 7 | **L-PFHxS** | NQ | NQ | NQ | NQ | 0.65 | 0.59 | 0.33 | NQ | NQ | NQ | 0.67 | 0.98 | NQ | 0.66 | NQ | *0.31* |
| 8 | **L-PFOS** | 1.23 | 1.80 | 0.23 | 2.12 | 0.53 | 3.90 | 1.37 | 1.66 | 2.01 | 2.44 | 0.50 | 0.35 | 1.71 | 3.73 | 0.44 | *0.19* |
|  | **Σ 8 PFASs** | **10.42** | **11.34** | **7.74** | **10.93** | **58.18** | **34.20** | **14.34** | **13.89** | **9.73** | **13.98** | **10.34** | **49.26** | **18.42** | **46.48** | **8.74** |  |
|  | **Σ PFCAs** | **9.20** | **9.54** | **7.51** | **8.81** | **57.00** | **29.71** | **12.63** | **12.23** | **7.72** | **11.54** | **9.17** | **47.93** | **16.71** | **42.09** | **8.30** |  |
|  | **Σ PFSAs** | **1.23** | **1.80** | **0.23** | **2.12** | **1.18** | **4.48** | **1.71** | **1.66** | **2.01** | **2.44** | **1.17** | **1.33** | **1.71** | **4.39** | **0.44** |  |

NQ- not quantified (concentration lower than MQL and treated as 0 in Σ8 PFASs, ΣPFCAs, ΣPFSAs)

**Table S2. Concentrations of PFASs in surface waters from Day river (ng/L) – the control area**

| **No** | **Name** | **R1** | **R2** | **R3** | **R4** | **R5** | **R6** | **R7** | **R8** | **R9** | **R10** | ***MQL*** |
| --- | --- | --- | --- | --- | --- | --- | --- | --- | --- | --- | --- | --- |
| 1 | **PFHxA** | 0.47 | 0.65 | 0.27 | 0.46 | 0.86 | 0.82 | 0.67 | 0.49 | NQ | NQ | *0.26* |
| 2 | **PFOA** | 1.51 | 1.05 | 2.01 | 1.61 | 1.43 | 1.59 | 0.95 | 1.17 | 0.55 | 1.33 | *0.35* |
| 3 | **PFNA** | 0.59 | 0.52 | 0.65 | 0.74 | 0.61 | 0.82 | 0.35 | 0.71 | 0.54 | 0.67 | *0.41* |
| 4 | **PFDA** | 0.89 | 0.89 | 0.79 | 0.61 | NQ | 0.62 | 0.65 | NQ | NQ | 0.62 | *0.36* |
| 5 | **PFUdA** | NQ | 0.43 | NQ | NQ | 0.37 | 0.32 | NQ | NQ | NQ | NQ | *0.49* |
| 6 | **PFDoA** | NQ | NQ | NQ | NQ | NQ | NQ | NQ | NQ | NQ | NQ | *0.29* |
| 7 | **L-PFHxS** | 2.82 | NQ | NQ | NQ | NQ | NQ | NQ | NQ | 1.86 | 1.49 | *0.31* |
| 8 | **L-PFOS** | NQ | NQ | NQ | NQ | NQ | NQ | NQ | NQ | NQ | NQ | *0.19* |
|  | **Σ 8 PFASs** | **6.28** | **3.56** | **3.71** | **3.42** | **3.27** | **4.16** | **2.63** | **2.36** | **2.95** | **4.11** |  |

NQ- not quantified (concentration lower than MQL and treated as 0 in Σ8 PFASs)
